# A Clinical Framework for Assessing Cannabis-Related Impairment Risk

**DOI:** 10.3389/fpsyt.2022.883517

**Published:** 2022-06-24

**Authors:** Caroline A. MacCallum, Lindsay A. Lo, Carly A. Pistawka, April Christiansen, Michael Boivin, Melissa Snider-Adler

**Affiliations:** ^1^Department of Medicine, Faculty of Medicine, University of British Columbia, Vancouver, BC, Canada; ^2^Department of Public Health Sciences, Dalla Lana School of Public Health, University of Toronto, Toronto, ON, Canada; ^3^Faculty of Science, University of British Columbia, Vancouver, BC, Canada; ^4^Centre for Neuroscience Studies, Queen's University, Kingston, ON, Canada; ^5^CommPharm Consulting, Barrie, ON, Canada; ^6^Department of Medicine, Faculty of Medicine, Queen's University School of Medicine, Kingston, ON, Canada

**Keywords:** cannabinoids, medical cannabis, THC, impairment, occupational safety, driving

## Abstract

Clinicians play an important role in promoting safe and responsible medical cannabis use. One essential component to safe use is considering a patient's risk of neurocognitive impairment. However, there remains a lack of practical guidance on how clinicians can evaluate this risk for medical cannabis patients. Here, a practical framework is presented for clinicians to assess and stratify cannabis-associated impairment risk. The proposed framework is intended to practically guide healthcare providers in gaining a more comprehensive review of a patient's impairment-related factors. This framework can be used to assess impairment risk for patients currently using or considering medical cannabis and is recommended for all patients who perform safety-sensitive duties. Healthcare providers (HCP) managing patient's medical cannabis or those conducting assessments to determine risk of impairment for safety-sensitive workplaces can utilize this framework to stratify patients' risk of impairment. Such assessments can inform patient-specific needs for support, education, and guidance, to ensure cannabis is used safely and responsibly.

## Introduction

As medical cannabis use increases worldwide, concerns have arisen over the potential for cannabis impairment during safety-sensitive work or activities (1). Currently, medical cannabis is most strongly indicated for chronic pain, spasticity associated with multiple sclerosis, chemotherapy-induced nausea and vomiting, and treatment of intractable seizures in Dravet and Lennox-Gastaut syndromes (2). Although evidence is less clear, medical cannabis is also commonly used to treat symptoms associated with neuropathic pain, fibromyalgia, arthritis, sleep disorders, anxiety, and depression (3–6). There are several routes of administration for cannabis, the most common for medical use are inhalation (e.g., smoking or vaporizing) and oral ingestion (e.g., oils or capsules) (7–9). Each route of administration has unique pharmacokinetic and pharmacodynamic properties, leading to different times of onset and duration of action (10, 11). Dosing and administration of medical cannabis is complicated by not only having multiple methods of administration, but also a wide variety of product types and chemovars. Cannabis products vary in their composition of the two primary cannabinoids, tetrahydrocannabinol (THC) and cannabidiol (CBD). Typically, cannabis treatment protocols are tailored to the individual patient, with the exact dose and administration protocol being dictated by patient-specific needs and goals of treatment (8). All of these factors influence the potential of cannabis-related impairment.

**Figure 1:**
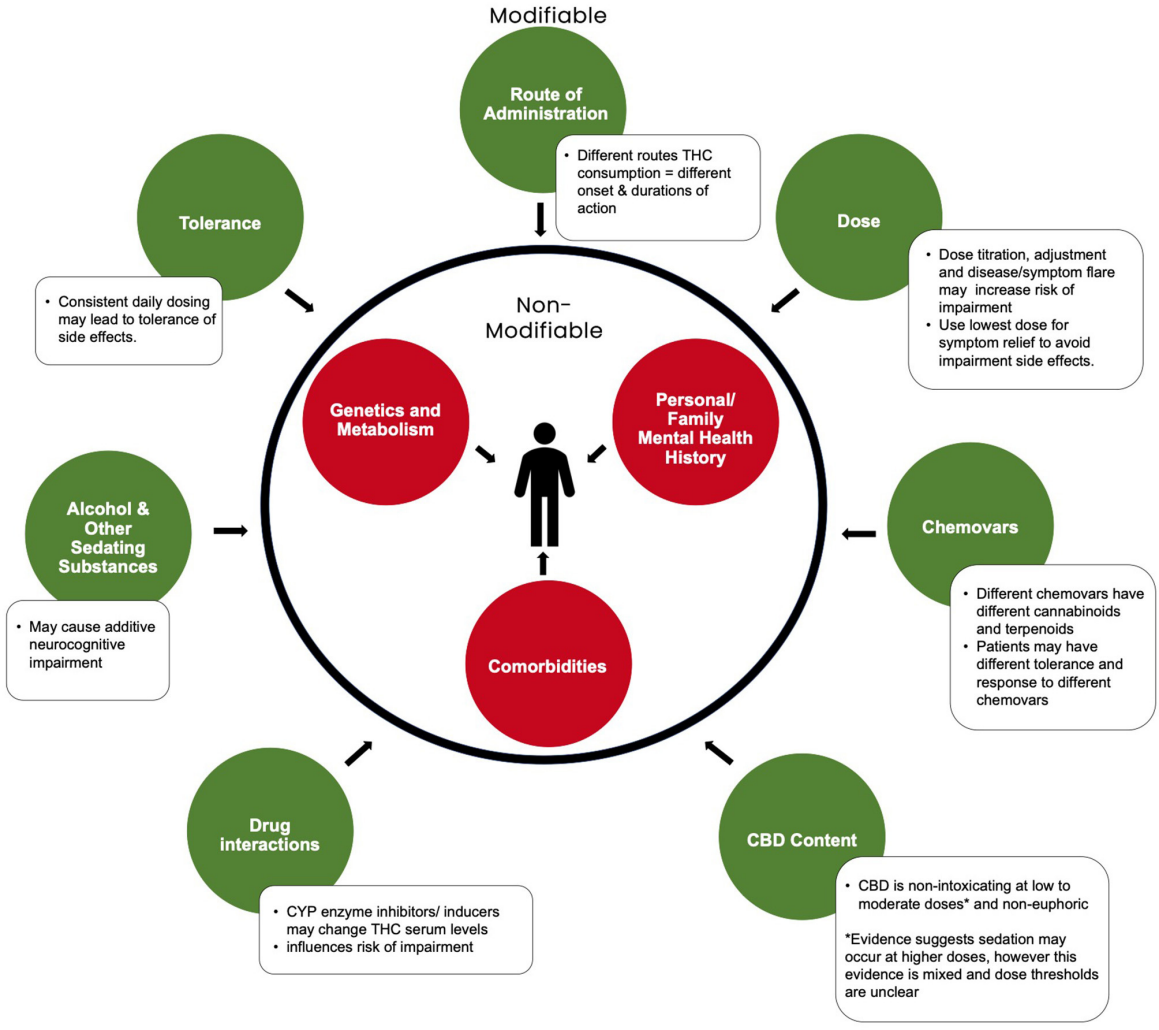
Modifiable and non-modifiable factors influencing cannabis-related neurocognitive impairment. Adapted from Eadie et al. (12)^2^.

Cannabis has the potential to impair multiple domains of neurocognitive function (12, 13). Evidence to date supports that THC is the primary psychoactive component in cannabis responsible for causing impairment (14). THC is a partial agonist for Cannabinoid receptor type 1 (CB1) and binds to CB1 receptors in regions of the brain involved with cognition, memory, anxiety, sensory perception, and motor coordination (15). This pharmacological action is what causes the dose-dependent disruption of cognitive and psychomotor domains important for safety-sensitive work or activities, such as driving motor vehicles (16, 17). In contrast, CBD, the other primary cannabinoid in cannabis, is generally considered non-impairing at low and moderate doses (See Figure 1) (18). Current evidence suggests CBD may cause sedation in some individuals at higher doses (19, 20). However, evidence is inconclusive and dose ranges are unclear. Some studies and reviews report no sedation at higher doses of 1,000–1,500 mg of CBD (11, 19, 21, 22), while others, primarily in pediatric epilepsy populations, report sedation at more moderate doses of 5–10 mg/kg/day CBD (20, 23, 24). Further investigation is needed to assess if there is a true dose-dependent effect or if sedation is due to the co-administration of other drugs such as antiepileptics or CNS depressants, which may lead to drug interactions resulting in increased sedation (20, 25, 26). As such, when discussing impairment there are a myriad of other factors that are important to consider beyond just the dose of THC that can contribute to an individual's risk (12).

Education and risk mitigation are important components of a clinician's role in promoting the safe and responsible medical cannabis use. Determining impairment risk has been a significant challenge for many clinicians. There is a lack of suitable testing metrics for determining cannabis impairment with a lack of established correlation between measurement of bodily fluids and level of impairment. Additionally, there is a lack of available well-rounded guidance or consensus recommendations to assess a patient's impairment risk. An additional challenge is the lack of literature available specifically focused on medical cannabis-related impairment. Here, we present a practical framework for clinicians to assess and stratify cannabis-associated impairment risk. Current evidence is interwoven within this practical framework.

## Framework for Assessing Impairment

This impairment framework has been developed to help guide healthcare providers (HCPs) assessing a patient's impairment risk (Table 1). The idea for this practical guide was born from a needs assessment conducted by author CM for continuing education programs, as well as recent published reports revealing a HCP need for practical guidance on assessing the many aspects of cannabis-related impairment (27, 28). This framework was developed through a combination of expert clinical opinion, reviewing common questions in medical education sessions conducted by the authors, and reviewing the available literature. The first step in developing this framework was translating the clinical processes used by authors CM, MB, and MSA when assessing patient impairment risk in-clinic into a step by step framework. The next step was a collaborative discussion reviewing common questions and points of concerns asked during medical education run by authors, these were then incorporated in the framework. A practical overview of the literature was then conducted to elaborate on each framework component and make final adjustments to content. Finally, author consensus based on expert clinical opinion and relevant literature categorized factors into higher, moderate, and lower risk of impairment. The outcome of this process resulted in a practical framework that can help guide clinicians when assessing their patients' potential risk of cannabinoid-related impairment. It is best practice to complete an assessment of impairment risk for patients being considered for or who are currently using medical cannabis, especially those in safety-sensitive occupations (e.g., driving, operating heavy machinery, dealing with hazardous materials, or working in a safety-sensitive workplace).

**Table 1:** Framework for assessing medical cannabis risk of impairment.

Cannabis initiation
How is the patient using or intending to use cannabis?
Cannabis product(s) being used
What are the methods of cannabis administration?
Is the cannabis source regulated, third party tested?
Dose, frequency, and length of use
What amount of THC and CBD is being used?
What is the frequency and time of day cannabis is being taken?
How long has the patient been stabilized on this dose and frequency?
Risk factors for impairment
Does the patient have any impairment-related adverse effects?
Are there patient factors that increase risk of impairment?
What other prescription or recreational drugs are being used?
Is the patient involved in a safety-sensitive occupation or duties?
How long between cannabis use and engaging in safety-sensitive activities?
Factors that may mitigate impairment
Does cannabis manage conditions that are associated with impairment?
Is the patient using CBD containing products?
Is there ongoing education and monitoring?

### Cannabis Initiation

#### How Is the Patient Using or Intending to Use Cannabis?

Clinicians should engage with their patients to understand the reasons why they are using cannabis. Medical and recreational cannabis have different goals of use (29, 30). In a strictly medical context, cannabis and certain cannabinoids are used to manage symptoms associated with a medical condition and improve an individual's ability to function (31). Patients with HCP authorizations for medical cannabis should have a formal diagnosis and documentation of their medical condition. In clinical settings, it has been observed that these patients typically titrate to the lowest dose required to obtain symptom relief, with acceptable side effects, and follow consistent and standardized dosing procedures (8). This pattern often leads to lower cannabinoid doses, thus reducing impairment risk and may support side effect tolerance development (8, 32). It is important to determine if cannabis was initiated by a knowledgeable, licensed HCP and if there is regular ongoing monitoring and support, as lack of education and guidance can increase the risk of misuse and possible impairment. Additionally, individuals reporting the use of medical cannabis, but are not under the guidance and monitoring of a knowledgeable HCP, may have use patterns more similar to recreational users (31).

Recreational cannabis is generally used by those seeking relaxation, euphoria and/or impairment. Recreational users often consume larger THC doses over a shorter period of time in order to obtain the desired effect. This pattern is associated with an increased risk of adverse effects and impairment (15, 33, 34). Recreational use also tends to be more inconsistent in product type and pattern of use (31, 35). This can lead to unpredictable effects, thus increasing the risk of impairment.

Some medical patients will also use cannabis recreationally. This too may increase risk of impairment as the effects and risks of THC are additive due to its highly lipophilic properties and accumulation of THC in adipose tissue (14). Clinicians are encouraged to approach the topic non-judgmentally. Consider one of the following approaches: “A number of my patients also use cannabis recreationally; do you use cannabis recreationally as well?” or “How often do you also use cannabis recreationally?”.

### Cannabis Product(s) Being Used

#### What Are the Methods of Cannabis Administration?

Different routes of cannabis administration have unique pharmacokinetic properties that dictate the duration of potential impairment and will when it is safe to engage in safety-sensitive activities (10, 36). It is important to understand the timeframe where a patient may be at risk in order to determine when cannabis can be used safely. Oral ingestion is a long-acting dosage form, with an onset of action within 1–2 h, lasting an average of 6–8 h (10, 37). Oral formulations are often ideal for medical use but there is also a greater period of potential impairment, and a risk for delayed impairment (38).

Inhalation is a short-acting dosage form, with an onset of 5–10 min, lasting an average of 1–4 h (14, 39). As a result, inhaled medical cannabis is commonly used for acute symptoms and presents a shorter period for potential impairment. However, there can be difficulties with accurate dosing, since length (time) and depth of inhalation significantly impact the cannabinoid dose consumed. This may increase risk of unintentional impairment.

We advise against the use of concentrated dosage cannabis forms for medical use (e.g., dabbing) as they are commonly associated with excessive impairment and health risks (40, 41). To date, local application of topical cannabinoids to intact skin does not appear to be associated with central effects, and thus can be used without risk of impairment (42).

#### Is the Cannabis Source Regulated, Third Party Tested?

Ensuring the cannabis product being used is from a regulated, third party tested supplier is important. Products from illicit sources may have mislabeled cannabinoid contents, presenting a risk of unexpected impairment. One study evaluating CBD products sold online, found that 21% of these products contained sufficient THC to produce impairment (43). Further, non-regulated products, especially purchased online, may contain synthetic cannabinoids or be more likely to be highly potent, increasing risk of impairment (40). Regulated products can provide some confidence that the label matches the product's cannabinoid content. Regulated products normally have strict regional requirements (state, provincial, or federal) for labeling and testing (40, 44).

### Dose, Frequency, and Length of Use

#### What Dose of THC and CBD Is Being Used?

Different chemovars (strains) will have different cannabinoid content. Cannabis dosing takes into consideration the THC and/or CBD content of each plant chemovar. In dried cannabis flower it is labeled as a percentage of cannabinoid in the total weight (%/g), or by concentration in cannabis oils (mg/ml). The majority of impairing adverse events are THC-dose dependent (12, 45). Of note, tetrahydrocannabinolic acid (THCA) is the carboxylic acid form of THC in the “raw” plant. THCA is non-intoxicating and non-impairing (46) unless decarboxylation through heating occurs (47, 48).

There is increasing evidence to support that CBD is non-impairing. High oral doses of 100 mg of CBD up to supratherapeutic doses of 1,500 and 4,500 mg of CBD have not produced detectable effects on cognitive or motor function (11, 21, 22).

Determining what THC dose will elicit impairment remains highly patient-specific, regardless of the method of administration. Given the multiple factors responsible for impairment (Figure 1), it is challenging to separate effects of THC dose, specifically in determining a “safe” dose that will be non-impairing for all patients. Experimental studies utilizing neuropsychological battery tests, simulator or on-road testing, were conducted to assess the influence of cannabis on driving, cognitive, and psychomotor ability. In healthy, infrequent cannabis users, acute oral THC doses of 7.5 and 15 mg slightly impaired time perception, therefore also affecting motor response preparation and execution processes, impulsivity and inhibition (49), as well as episodic memory and learning (50). However, these same doses did not significantly alter performance on the Digit Symbol Substitution Test, Hopkins Verbal Learning Task, Digit Span Forward, Go/no-go, or the Delay or Probability discounting tasks (49). Other studies report that relative to placebo, 10 mg of oral THC did not appear to alter cognitive or psychomotor performance among healthy, infrequent cannabis users (51). Importantly, participants of these studies would not have been on stable doses of medical cannabis. A recent randomized, controlled trial found low, single doses of 0.5–1.0 mg inhaled THC did not result in impairment in processing speed (Reaction Time Test, RTI), episodic memory (Paired Associates Learning Task, PAL), working memory (Spatial Working Memory Test, SWM) or sustained attention (Rapid Visual Information Processing Test, RVP) in patients with chronic pain (52). While doses above 40 mg of THC are considered high and carry a substantial risk of impairment (32, 37). The risk of impairment for doses between these ranges strongly depends on patient-specific factors. In alignment with previous literature (53), we believe *stable* doses below 10 mg/day generally carry a lower risk of impairment.

For dried product, evidence supports that most medical cannabis patients have therapeutic benefit from between 1 and 3 g of cannabis per day (44). Consuming over 5 g/day of dried cannabis flower is a potential flag of problematic use (37). Problematic use is associated with a high risk for cannabis impairment and should be intervened for a variety of health-related reasons.

#### What Is the Frequency and Time of Day Cannabis Is Being Taken?

Frequency and pattern of use are important in determining the total daily dose and the times of the day for which a patient may be at the highest risk of impairment. Greater frequency of use results in longer periods of potential impairment and less time between cannabis use and engaging in driving or safety-sensitive duties. Daytime THC use may present a greater safety risk, especially if the patient engages in safety-sensitive activities during the day. The pattern of use will depend on patient-specific goals. Assessing the timeframe between use of cannabis and driving or engaging in safety-sensitive positions/workplaces is imperative when assessing risk. If the frequency of use is such that an individual is using inhaled cannabis within 4–6 h prior to driving or 8–12 h prior to engaging in safety-sensitive positions/workplaces respectively, then the individual would be considered higher risk based on the frequency and time of day cannabis is taken. Given the longer duration of action of orally ingested cannabis, longer timeframes are recommended (Table 3).

#### How Long Has the Patient Been Stabilized on This Dose and Frequency?

As with any pharmacotherapy, periods of medication titration or dose adjustment increases the risk of adverse events. Chronic and continuous medical cannabis use can lead to tolerance to many potential adverse side effects such as fatigue, dizziness, and acute intoxication (54). This is similar to other prescription medications used in this patient population.

A recent systematic review and meta-analysis found that regular cannabis users experienced less impairment in discrete driving-related cognitive skills compared to occasional users following acute consumption of a single dose of THC (~20 mg) (55). Other studies have corroborated these findings, reporting that frequent cannabis users (smoking ≥ 4 days/week) demonstrated less acute impairment across several neuropsychological tests compared to occasional users (smoking ~1 day/week) as a potential consequence of tolerance (56). However, another recent systematic review of meta-analyses concluded that acute and non-acute, residual impairment (within minutes to hours post-acute intoxication phase) in executive function, processing speed, verbal learning and memory, and attention may occur with regular, mostly heavy, consumption despite potential tolerance (13). It is important to note that this low-to-moderate quality evidence was extracted from a heterogeneous group of studies which varied in the operationalization of cannabis use history (frequency), cognitive tests used, cannabis dose, and control variables employed. As evidence is still varied on whether regular consumption of cannabis can lessen the risk of acute impairment as a result of developed tolerance, it cannot be assumed that patients frequently using cannabis, even at medically appropriate doses, are not at risk of impairment.

Clinicians should actively discuss dose stability with patients to determine if tolerance is developing. HCPs should be cautious in recommending safety-sensitivity activities even in a patient with potential tolerance. Tolerance to cannabis, as with other substances, may not equate to complete lack of impairment.

### Risk Factors for Impairment

#### Does the Patient Have Any Impairment-Related Adverse Effects?

Adverse effects are a common sign of an excessive cannabis dose. Common cannabis-related impairment adverse effects are not experienced by the majority of patients using medical cannabis when the THC starting dose is low and titration is slow. The presence of certain adverse effects may result in impairment (Table 2). Generally, if a patient experiences these adverse effects, safety-sensitive activities should be refrained from and adjustments to the cannabis regimen are recommended.

**Table 2:** Adverse effects that may be associated with an increased impairment risk (9, 16).

**Impairment-related adverse effects**
**Neurocognitive**
• Cognitive effects (e.g., impaired short-term memory, decision-making, decreased concentration, divided attention)
• Dizziness
• Drowsiness
• Fatigue
**Sensory-perceptual**
• Ataxia or discoordination
• Blurred vision
• Headache
**Mental health**
• Anxiety
• Euphoria
• Psychosis/ paranoia
**Cardiovascular**
• Orthostatic hypotension
• Tachycardia (if results in anxiety, dizziness, syncope, or myocardial infarction)
Gastrointestinal
• Cannabis hyperemesis syndrome

#### Are There Patient Factors That Increase Risk of Impairment?

Patients with comorbidities that result in fatigue, dizziness, or cognitive slowing may compound impairment (8, 12). Notable conditions to consider include, but are not limited to, older age, concurrent mental health conditions, substance use disorders, neurodegenerative disorders, sleep disorders, and chronic pain conditions (8, 57–59). These conditions alone, and in combination with cannabis, may impair an individual's ability to be alert and engage in normal cognitive or motor function. Additional patient factors that are important to consider are concurrent medications and driving/safety-sensitive occupations, which are discussed below (8, 12, 58, 59). Patients with factors that may cause additive impairment should be monitored more closely to ensure absence of adverse effects.

#### What Other Prescription or Recreational Drugs Are Being Used?

Drug interactions may increase risk of impairment. Medical cannabis patients commonly take other impairing medications to manage their condition(s). While cannabis is believed to be safe to use with most medications, clinicians should assess all other medications for potential interactions (60). Common prescription or over-the-counter medications that may pose a risk for additive impairment or sedation when combined with THC include antiepileptics, antipsychotics, benzodiazepines, opioids, tricyclic antidepressants, dimenhydrinate, diphenhydramine, or muscle relaxants (61). The use of recreational substances such as alcohol as well as other illicit substances can also cause increased impairment.

Since cannabis is metabolized in the liver by CYP 450 isoenzymes (THC: CYP2C9, CYP2C19, CYP3A4, and CBD: CYP2C19, CYP3A4), CYP inhibitors or inducers may cause pharmacokinetic drug interactions, which can impact the blood serum levels of cannabinoids or the interacting medication (61). It should be noted that there is an indirect potential for impairment with moderate to high doses of CBD when taken with other CYP3A4 inhibitors (e.g., anti-seizure medications such as clobazam) (62). Additionally, drug interactions that increase or prolong the availability of THC may lead to prolonged impairment. In patients with potential drug interactions, increased monitoring and drug levels, when appropriate, should be carried out until absence of impairment or adverse effects are ruled out.

#### Is the Patient Involved in a Safety-Sensitive Occupation or Duties?

The patient's specific lifestyle should be considered when determining risk of impairment. If a patient does not drive or work in a safety-sensitive position or workplace, the outcomes of impairment are generally less serious. Safety-sensitive activities can include such tasks as operating transportation, use of heavy machinery, and dealing with hazardous materials. The consequences of even mild impairment can be more profound in these circumstances, impacting the worker, their colleagues, the community, and the environment. Extra precaution and focus on mitigating impairment risk should be taken for those who work in a safety-sensitive position or workplace.

#### How Long Between the Use of Cannabis and When the Patient Engages in Safety-Sensitive Activities?

Although driving a personal motor vehicle is considered a safety-sensitive activity, those who work in safety-sensitive occupations, where impairment may lead to catastrophic consequences in the workplace, may require more stringent restrictions in dose and timing of administration of cannabis. The more complex the task, the less likely individuals can compensate for the mild to moderate impairments associated with cannabis use. Due to the significant hazard associated with any impairment, tighter restrictions for those in safety-sensitive occupations should be considered and an abundance of caution is reasonable and recommended (63).

Regarding driving a car, a patient is generally considered low risk when driving the morning after inhaling a stable dose of THC the previous evening. Educating patients on windows of impairment in which driving should be avoided is critical. The 2021 Canadian Cannabis Survey revealed that 21% of people reporting cannabis use in the last 12 months had driven within 2 h of smoking or vaporizing. Of individuals reporting driving within 2 h, 78% reported they did not feel impaired and 22% reported that they thought they could drive carefully (64). This highlights the importance of HCP guidance to mitigate potential harms.

It is important to know the route of administration as each has a different duration of action and periods of potential impairment. This should be considered in the context of when cannabis is being used and when an individual is safe to operate a motor vehicle or performs any safety-sensitive duty.

A review containing six RCT's in medical cannabis populations found impairment resolved within 2–4 h post dose,^2^ in line with several other clinical trials (56, 65, 66). However, until there is more robust literature for medical cannabis populations, a cautious approach of consuming THC at least 4–6 h, if inhaled, and 6–8 h, if ingested, prior to operating a personal motor vehicle is suggested (6, 29).

Longer duration between timing of dose and the start of work, as well as tighter restrictions on dosing of THC may be required for patients who work in a safety-sensitive position or workplace. We advise waiting at least 8–12 h, if inhaled, and 12 h, if ingested, prior to engaging in safety-sensitive positions or workplaces.

### Factors That May Mitigate Impairment

#### Does Cannabis Manage Conditions That Are Associated With Impairment?

Certain medical conditions can increase the risk of impairment. Studies have shown conditions such as multiple sclerosis, insomnia, epilepsy, anxiety, and depression have an increased risk of motor vehicle accidents (67–69). Reducing or eliminating the symptoms associated with these medical conditions can therefore decrease risk of impairment. If medical cannabis is successful in controlling symptoms that may impact motor or cognitive function on their own, individuals may actually have a lower risk of impairment (70).

#### Is the Patient Using CBD Containing Products?

Evidence is still varied on whether or not CBD can lessen the impact of THC-associated side effects (71), but using products that contain CBD may allow for a reduced THC dose required due to synergistic effects (72). THC and CBD combinations were also associated with positive effects on symptoms, while experiencing significantly less paranoia and anxiety than THC-only products (72). From a clinical and safety standpoint, CBD is a preferred choice for individuals that engage in safety-sensitive activities. It is important to note that many CBD-dominant products still contain low levels of THC.

#### Is There Ongoing Education and Monitoring?

Many individuals consume medical cannabis without proper safety education (73). As per best practice standards, HCPs should provide education on side effects, product/chemovar selection, activity limitations, dosing and titration, method of administration, and treatment monitoring to reduce the risk of patient harm (8, 32). The frequency of monitoring will depend on patient specific circumstances, clinician experience, and guidelines by local regulatory bodies. HCPs are advised to tailor the frequency of monitoring to reflect the benefit and risk considerations for the individual patient.

## Discussion

The lack of suitable testing metrics poses a challenge in determining cannabis-related impairment. The proposed framework is intended as a practical guide for HCP's to comprehensively assess and stratify the potential risk of impairment in their patients. This information guides discussion and patient education regarding these potential risks and allows for adjustments to mitigate or reduce the risk of impairment. This is especially important for individuals who perform any safety-sensitive activities.

Whether it be returning to work, driving, or working in a safety-sensitive position or workplace, the potential for cannabis impairment should be evaluated. Factors associated with different levels of impairment risk are summarized in Table 3. To stratify risk for any patient, each factor must be considered and assessed. If any considerations fall under higher risk for impairment, the individual is considered higher risk, regardless of the number of risk factors in the moderate or lower risk of impairment categories. Similarly, if any considerations fall under moderate risk, with no higher risk of impairment considerations, the individual is considered at moderate risk of impairment. An individual can only be considered to be at lower risk of impairment if all considerations fall under the lower risk category.

**Table 3:** Factors to consider when assessing impairment risk (9, 10, 43, 50).

**Consideration**	**Factors associated with a higher risk of impairment**	**Factors associated with a moderate risk of impairment**	**Factors associated with a lower risk of impairment**
**Cannabis initiation**	**Not** initiated on medical cannabis by a HCP Patient is **not stabilized** on cannabis	Initiated by a HCP with **limited knowledge** of medical cannabis Patient has recently initiated cannabis or is still titrating dose of cannabis (**not stabilized)**	Initiated by a HCP **knowledgeable** in cannabinoid medicine Cannabis is used for a specific medical condition or symptoms *Patient has been **stabilized** on cannabis for **at least 2 weeks**
**Product info**	Products are **not** purchased from a regulated, third party tested supplier (19)	**Not all** products are purchased from a regulated, third party tested supplier (19)	All products are purchased from a regulated, third party tested supplier (19)
**THC dosage**	*Cannabis use includes use of **high dose THC** (above 40 mg THC/day) or use of cannabis concentrates (including dabbing)	*THC dosing **above 10 mg** THC/day but below 40 mg THC/day (9, 10)	*THC dosing **<**10 mg of THC/day Those working in safety-sensitive positions or workplaces may require even lower THC daily dose.
**Restriction period**	*Inhaled products: <2 h prior to driving *Inhaled products: <8–12 h after inhaling cannabis products for those in safety-sensitive positions/workplaces *Oral ingestion: <4 h prior to driving *Oral ingestion: <12 h after ingesting cannabis products for those in safety-sensitive positions/workplaces	*Inhaled products: <4–6 h prior to driving *Oral ingestion: <6–8 h prior to driving	**Inhaled products:** 4–6 h prior to driving (43). *Inhaled products: At least 8–12 h after inhaling cannabis products for those in safety-sensitive positions/workplaces **Oral ingestion:** 6–8 h prior to driving (43). *Oral ingestion: At least 12 h after ingesting cannabis products for those in safety-sensitive positions/workplaces Localized topical cannabis may be used on intact skin due to limited systemic absorption
**Adverse events**	*Reports **multiple** impairment related adverse effects of moderate to severe intensity	*Reports **one** impairment- related adverse effects of mild intensity	*Reports **no** impairment-related adverse effects
**Concurrent medications and comorbidities**	Patient has comorbidities associated with impairment (9, 50) *Using **≥2** other medications that may be impairing or result in additive sedation or adverse events	Patient has comorbidities that **may** increase risk of impairment (9, 50) *Using one medication that may be impairing or result in additive sedation or adverse events	Patient **does not** have any other comorbidities that increase risk of impairment (9, 50) *No use of other medications that may be impairing or result in additive sedation or adverse events
**Recreational substance use**	Patient **regularly** uses other recreational substances including recreational cannabis	Patient **occasionally** uses other recreational substances including recreational cannabis	Patient **does not** use any recreational substances including cannabis
**Education and monitoring**	**Not** monitored by a HCP (9). Only cannabis education was acquired from non-HCP sources	Monitored by a HCP with **limited knowledge** of medical cannabis (9) Basic education from HCP on safe medical cannabis use	Monitored by a HCP **knowledgeable** in cannabinoid medicine (9). Advanced HCP education on safe medical cannabis use

*Based on authors evidence-informed expert opinion.

The framework presented in this piece is intended as a proposed guide to help clinicians assess risk of cannabinoid-related impairment in their patients. However, it is not without limitations. Although the framework discussed is commonly used in-clinic by authors, it has not been formally evaluated. Thus, we cannot formally speak to its reliability or validity. Despite this, the current lack of available guidance on the topic gives merit to share available guidance while more standardized processes are developed. Second, cannabis-related impairment is a complex topic, as there is a wide range of domains through which impairment may occur and there is notable variability between patients. While this framework is meant to provide a general overview, it should not be forgotten that each patient requires an individualized assessment and may have unique factors that influence impairment risk. Third, using this framework relies on patients providing honest and complete information. Without this, the guidance could be misinformed and could cause liability for HCPs and those relying on the risk assessment (employers for example). This stresses the importance of developing good rapport and trust with the patient to promote open and honest conversation. Additionally, taking the time to educate the patients on the danger of engaging in safety sensitive activities or work and how to mitigate this risk is key.

Future directions in this work should look at the reliability and validity of this framework more formally. Developing a points system may be a useful avenue to pursue to help consider all risk factors more clearly. Medical cannabis patients are a heterogenous population, thus another avenue would be investigating how cannabis-related impairment differs between medical populations, and if there are differing key factors that may promote or mitigate impairment.

## Conclusion

Factors discussed in the framework can impact the degree and duration of impairment. Although this framework is guided by the current evidence, more research in this area can provide stronger guidance on potential risk factors for cannabis-related impairment. Each patient will have unique considerations. Proper screening and evaluation of a patient can help promote the safe and responsible use of medical cannabis.

## Author Contributions

CM was primarily responsible for the conceptualization and overall intellectual leadership of this project. In collaboration with CM, LL wrote the first draft of this manuscript with additional support from CP. AC, MB, and MS-A contributed to revising the manuscript with additional intellectual input. All authors contributed to the article and approved the submitted version.

## Conflict of Interest

CM is the Medical Director of Greenleaf Medical Clinic and Chief Medical Officer for Translational Life Sciences. She is on the Board of Directors for The Green Organic Dutchman. She is an advisor to PreveCeutical, Pinnacle Care, Africanna, EO Care, Andira Medicine, Active Patch Technologies, Syqe Medical, and Dosist. Additionally, she has provided medical consultation and/or received support for industry sponsored continuing medical education from: Aleafia, Aurora, Canopy, Spectrum, Tilray, Emerald Health, and CCRN. MB has received financial support as a speaker and consultant for CHE activities from Teva, Pfizer, Novo Nordisk, Khiron, Tilray, mdBriefcase, J&J, Abbvie, Ascensia, Astra Zeneca, Biosynt, and Emergent BioSolutions. MS-A has received financial support from Syqe Medical and is on the advisory board of Syqe Medical and Indivior Canada. The remaining authors declare that the research was conducted in the absence of any commercial or financial relationships that could be construed as a potential conflict of interest.

## Publisher's Note

All claims expressed in this article are solely those of the authors and do not necessarily represent those of their affiliated organizations, or those of the publisher, the editors and the reviewers. Any product that may be evaluated in this article, or claim that may be made by its manufacturer, is not guaranteed or endorsed by the publisher.
